# United States Sanitary Commission

**Published:** 1864-01

**Authors:** 


UNITED STATES SANITARY COMMISSION,

*To His Excellency the President of the United States:*

Sir:—The United States Sanitary Commission authorized by the government to act as a Commission of Inquiry and Advice in respect to the Sanitary interests of the National Forces, have been for more than two years and a half close and careful students of the medical and hygienic affairs of the army. They ought to be, they are thought by the people of the United States to be, they claim to be, better acquainted with the working of the Medical Department, whose deficiencies, mistakes, and necessities, it is their solemn duty to discover and obviate, than any other responsible body of witnesses. Trusting in their discretion, zeal, and works, the people of the loyal States have made them their almoners, to the extent of seven millions of dollars worth of Sanitary Stores, and a million of dollars in money. The disbursement of this immense charity has brought our agents into close and continual contact with the Medical Department, to whose steady and rapid improvement from the imperfect state in which we found it, to its present degree of surprising and gratifying efficiency, we are able to lend a most indisputable testimony. We attribute this immense improvement to the fact that for two years the Medical Department has been directed by Dr. W. A. Hammond, Surgeon-General; a man known to all impartial and competent judges, as thoroughly scientific, highly endowed, large-minded, and an energetic and controlling administrator. He was selected for his office solely for fitness, and in our calm and deliberate judgment, his administration has more than justified all the high hopes and expectations of those who recommended him for the place.

We hear from sources that do not permit us to doubt the fact, that cautious but systematic efforts are now making to remove Surgeon-General Hammond from office. In the name of some millions of constituents, in the name of the homes of this country, whose solicitude, liberality, and watchfulness, we represent, we respectfully and conscientiously protest against the secret tribunal, and the indirect methods, by which the good fame of the Surgeon-General has been already seriously, and we believe unjustly, aspersed. We protest, in our character of experts, a body whose business it has been made, to inquire and advise on this very subject, that the removal of Dr. Hammond would be as serious a blow, at the lives, comfort, and efficiency of the army, as the enemy itself could inflict; that the science of the country, the humanity of its homes, and the army itself, would resent it, as a cruel wrong and an alarming error; and we feel ourselves bound, in the interests of the Soldiers in the field, and of those about to enter the service of the country, in the defence of our own principles and convictions, and in the name of the science, the charity, and the fair-mindedness of the Nation, to beg that no further steps in this direction may be taken, without a full and fair trial of Surgeon-General Hammond upon the charges alleged to have been secretly made against him.

December 29, 1863.

H. W. Bellows, Wolcott Gibbs,
Wm. H. Van Buren, Geo. T. Strong,
C. R. Agnew,
Standing Committee U. S. Sanitary Commission.

—[*New York Medical Times.*

